# Artificial Intelligence-Driven Telehealth Framework for Detecting Nystagmus

**DOI:** 10.7759/cureus.84036

**Published:** 2025-05-13

**Authors:** Harshal Sanghvi, Ali A Danesh, Jillene Moxam, Sandeep K Reddy, Gurnoor S Gill, B. Sue Graves, Sajeel Chowdhary, Kakarla Chalam, Shailesh Gupta, Abhijit S Pandya

**Affiliations:** 1 Department of Information Technology and Operations Management, College of Business, Florida Atlantic University, Boca Raton, USA; 2 Department of Communication Sciences and Disorders, Florida Atlantic University, Boca Raton, USA; 3 Department of Technology and Clinical Trials, Advanced Research, Deerfield Beach, USA; 4 Department of Electrical Engineering and Computer Science, Florida Atlantic University, Boca Raton, USA; 5 Department of Medicine, Charles E. Schmidt College of Medicine, Florida Atlantic University, Boca Raton, USA; 6 Department of Exercise Science and Health Promotion, Florida Atlantic University, Boca Raton, USA; 7 Department of Neurology, Baptist Health Medical Center, Boca Raton, USA; 8 Department of Ophthalmology, Loma Linda University Medical Center, Loma Linda, USA; 9 Department of Ophthalmology, Broward Health North, Deerfield Beach, USA

**Keywords:** diagnostic testing, evaluation for telehealth, nystagmus, ophthalmic research, ophthalmology

## Abstract

Purpose: This study reports the implementation of a proof-of-concept, artificial intelligence (AI)-driven clinical decision support system for detecting nystagmus. The system collects and analyzes real-time clinical data to assist in diagnosing, demonstrating its potential for integration into telemedicine platforms. Patients may benefit from the system’s convenience, reduced need for travel and associated costs, and increased flexibility and increased flexibility through both in-person and virtual applications.

Methods: A bedside clinical test revealed vertigo during rightward body movement, and the patient was referred for videonystagmography (VNG). The VNG showed normal central ocular findings. During the right Dix-Hallpike maneuver, the patient demonstrated rotatory nystagmus accompanied by vertigo. Caloric tests revealed symmetric responses, with no evidence of unilateral or bilateral weakness. A cloud-based deep learning framework was developed and trained to track eye movements and detect 468 distinct facial landmarks in real time. Ten subjects participated in this study.

Results: The slow-phase velocity (SPV) value was verified for statistical significance using both VNG machine-generated graphs and clinician assessment. The average SPV was compared to the value generated by the VNG machine. The calculated statistical values were as follows: p < 0.05, a mean squared error of 0.00459, and a correction error of ±4.8%.

Conclusion: This deep learning model demonstrates the potential to provide diagnostic consultation to individuals in remote locations. To some extent, it may supplement or partially replace traditional methods such as VNG. Ongoing advancements in machine learning within medicine will enhance the ability to diagnose patients, facilitate appropriate specialist referrals, and support physicians in post-treatment follow-up. As this was a proof-of-concept pilot study, further research with a larger sample size is warranted.

## Introduction

Historically, clinical medicine and engineering have been distinct disciplines. However, recent advances in artificial intelligence (AI) have driven significant interdisciplinary collaboration, particularly in healthcare [1]. Specialties such as ophthalmology [2,3], radiology [4,5], and pathology [6,7], which heavily rely on medical imaging, have seen notable benefits. AI enables the development of new diagnostic models that improve efficiency and accuracy by leveraging electronic health data [8].

Audiology has also benefited, with machine learning and neural networks enhancing hearing aids and tailoring experiences for patients [9]. AI-driven diagnostic models now assess disease severity via medical imaging, aiding physician decision-making and monitoring disease progression [10-12]. Despite these advancements, few studies have explored deep learning models that utilize real-time data. This study addresses this gap by evaluating a deep learning model designed to capture real-time data for diagnosing nystagmus, highlighting its potential as a tool for clinical decision support.

Nystagmus is characterized by involuntary, rhythmic eye movements often associated with vestibular or neurological disorders. It can result in symptoms like vertigo, dizziness, imbalance, blurry vision, and oscillopsia [13-17]. Eye movements may be vertical, horizontal, torsional, or a combination of these. Jerk nystagmus, marked by a slow defoveating phase followed by a rapid refoveating phase, is typically linked to vestibular dysfunction. In contrast, pendular nystagmus, which involves slow, equal-velocity movements in both directions, is more commonly associated with neurological conditions [14,15]. Acquired nystagmus is of particular clinical concern due to its association with conditions such as multiple sclerosis, stroke, vestibular imbalance, trauma, substance use, and adverse effects of medications [17-19].

Current clinical methods such as videonystagmography (VNG) [20] and electronystagmography (ENG) [21] are considered gold standards for diagnosing nystagmus. However, both have notable limitations. ENG is susceptible to artifacts and has difficulty detecting torsional eye movements, while VNG, though more accurate, is costly (approximately $250,000), requires substantial space, and may cause patient discomfort [22-25].

Recent advances in AI aim to address the limitations of traditional diagnostic methods. For instance, Wagle et al. [26] developed the aEYE model, which utilizes video oculography to screen for nystagmus. Other approaches by Punuganti et al. [27] and Wei et al. [28] focus on detecting quick and slow phases of eye movements. Bastani et al. [29] and Friedrich et al. [30] have further advanced AI-driven smartphone and webcam applications for remote diagnosis. The integration of AI with telemedicine offers cost-effective and accessible diagnostic solutions, particularly valuable for underserved populations [31-39]. For example, von Martial et al. implemented a tele-neurology model in emergency departments, reducing patient wait times and improving access to specialist care [33].

This study evaluates an AI-driven telehealth framework for real-time nystagmus detection, comparing its accuracy with that of VNG. The proposed model aims to improve accessibility, reduce costs, and enhance diagnostic efficiency, especially in remote settings. We hypothesize that our AI-driven telehealth framework, which utilizes smartphone-recorded videos and cloud-based analysis, will achieve comparable or superior accuracy to traditional VNG in measuring slow-phase velocity (SPV) while significantly increasing accessibility and affordability for clinicians and patients.

The model identifies 468 facial landmarks from video recordings, calculates eye movement speed, and generates visual graphs for physician interpretation. Physicians review these automated analyses to guide patient management decisions, which are documented in electronic health records (EHRs) and communicated to patients. This approach integrates AI diagnostics seamlessly into clinical workflows, reducing dependence on costly equipment and improving diagnostic access and efficiency. Ultimately, the combination of AI, cloud analytics, and telemedicine provides an innovative solution for diagnosing nystagmus, enhancing early detection and intervention, particularly in areas with limited access to specialized neuro-ophthalmologic care.

## Materials and methods

Framework

Deep learning can provide new accessible methods of determining nystagmus in individuals by creating less expensive, convenient models and implementing them directly into traditional clinical settings or a broader horizon of at-home telehealth patient visits, such as through telemedicine. With the rapid advancements in telemedicine and its integration into the US healthcare system, the pairing of AI and telemedicine can assist in addressing existing inequities [30-38] and potentially decrease the burden on the healthcare system, especially in rural and other underserved areas. This pairing can provide helpful information to physicians during at-home telehealth visits when treating patients. von Martial et al. developed a model for use in emergency departments, enabling tele-neurologists to diagnose nystagmus patients [33]. This method is essential in limiting patient wait times in emergency departments and getting them to the correct specialist for treatment [33]. 

Table 1 shows a detailed overview of the frameworks that the other authors have developed.

**Table 1:** Comparison of the proposed framework with other methods

Reference	Method used	Dataset utilized	Results	Summary
Proposed method	Train-test- validate = 70:20:10	15,000 frames	Accuracy = 98%, p < 0.05, mean squared error = 0.00459, correction error = ±4.8%	The authors have developed a telemedicine-assisted framework that allows caretakers to record videos of a patient's eye movement and upload them to a software for analysis. The system calculates beats per second and generates a visual graph, which is then reviewed by a physician. Based on this evaluation, appropriate management recommendations are made.
Winnick et al. [40]	Train-test = 80:20	24,521 nystagmus videos	Accuracy = 96.10%, specificity = 94.60%, sensitivity = 91.20%	Experiments indicate that the designed method can effectively identify torsional nystagmus. Compared with other methods, it has high recognition accuracy.
Reinhardt et al. [41]	Train-test = 80:20	1,090 patient videos	Accuracy = 91%	A vertical nystagmus recognition method is proposed based on deep learning. Using the same training dataset and test set, the recognition accuracy of this method for vertical nystagmus was 2% higher than other methods.
Yiu et al. [42]	Train-test = 7:3	728 nystagmus videos	Accuracy = 94.91%	Proposed a nystagmus video classification network (NVCN) to categorize nystagmus patterns. The experimental results prove that our proposed framework can recognize different nystagmus patterns effectively.
Pham et al. [43]	Train-validate-test for cross-validation = 7:1:2	8,284 frames	Accuracy = 99%	A convolutional neural network‐based nystagmus extraction system, ANyEye, optimized for videonystagmography data is proposed.
Rosengren et al. [44]	Train-validate- test = 304:93:122	3,496 horizontal nystagmus and 5,962 vertical/torsional nystagmus of 854 patients	Accuracy = 88.45%, specificity = 88.41%, sensitivity = 88.48%, F1 sore = 89.14%	Convolutional neural network (CNN)-based models were trained to detect nystagmus patterns in 26 three dimensions (horizontal/vertical/torsional) and evaluate the performance of the BPPV diagnosis system.
Banoub et al. [45]	Clinical investigation	17 healthy subjects induced nystagmus on eight occasions over 30 days	Accuracy = 99.10%, specificity = 98.60%, sensitivity = 99.10%	The continuous ambulatory vestibular assessment (CAVA) device has been developed to continuously monitor eye movements, allowing insight into the physiological parameters present during a dizziness attack. The device operated effectively as an ambulatory monitor, allowing the reliable detection of artificially induced nystagmus.
Gajjar et al. [46]	Train-test = 60:40 (slow-phase algorithm)	524 VOG traces (262 horizontal traces, 262 vertical traces)	Specificity = 84%, sensitivity = 78.80%	Video-oculography (VOG) plots were analyzed from consecutive patients with dizziness presenting to a neurology clinic to verify the accuracy of automated nystagmus detection algorithms. Results determined that slow-phase and fast-phase algorithms were accurate for detecting nystagmus, and combining algorithm parameters using logistic regression models mildly improved detection accuracy, indicating that machine learning may potentially optimize the accuracy of future automated nystagmus detection systems.
Patel et al. [47]	Train-test = 3:1	27,535 labeled images	Accuracy = 62%, sensitivity = 90%, F1 score = 63%	A novel location- and time-independent mobile application for VNG to support vertigo patients and medical staff.
Alhalabi et al. [48]	Train-test	3,946 eye image frames	Accuracy = 93%, specificity = 93%, sensitivity = 94%	Accurate pupil localization is a prerequisite for many eye-tracking and video-oculography (VOG) methods. Proposed to use a fully convolutional neural network (FCNN) for segmentation of the whole pupil area, trained on 3,946 VOG images.
Alhalabi et al. [49]	Train-validate = 668:78	746 patient videos	Accuracy = 91%, F1 score = 90%	‘‘Look and diagnose’’ (LAD), a hybrid deep learning-based system that aims to support doctors in the medical field in effectively diagnosing benign paroxysmal positional vertigo (BPPV) disorder.

Table 1 summarizes various methods and studies related to detecting and classifying nystagmus using machine learning and telemedicine tools. It highlights the training, testing, and validation ratios, datasets used, key performance metrics, and unique contributions of each approach. The results showcase a range of techniques, including convolutional neural networks (CNNs), video-oculography (VOG), and hybrid deep learning systems, achieving high accuracy and sensitivity in nystagmus detection. These studies demonstrate advancements in telemedicine and automated systems to assist healthcare providers in effectively diagnosing and managing conditions like benign paroxysmal positional vertigo (BPPV) and other vestibular disorders.

**Figure 1:**
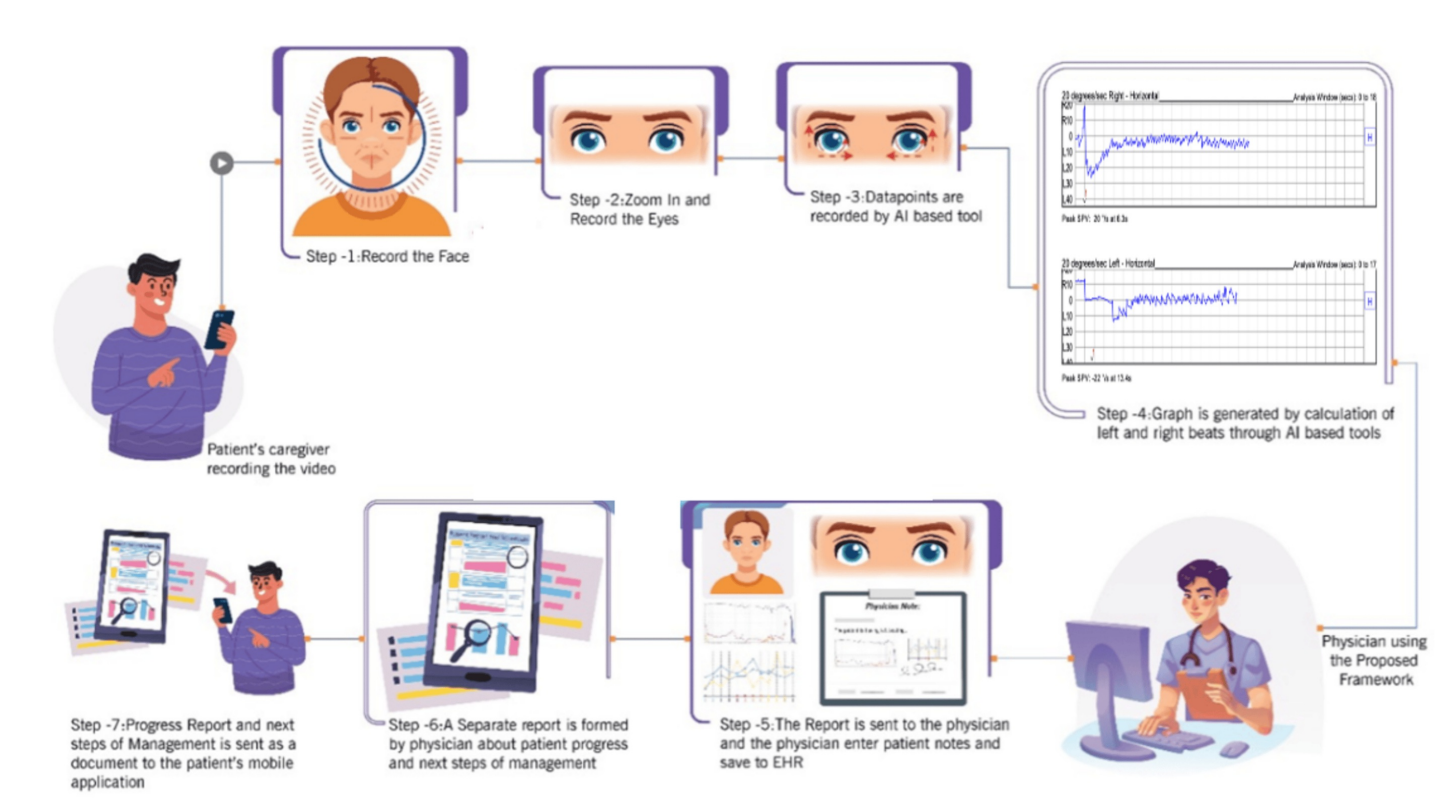
Methodology for the proposed AI-driven framework for detecting nystagmus through an AI-based telehealth framework Image Credits: Vidhi Patel. This image was created using PowerPoint (Microsoft Corp., Redmond, WA, US).

Figure 1 demonstrates the framework of the proposed clinical decision support system. A patient's caregiver begins by using a smartphone or similar device to record a video of the patient's face. The process involves zooming in on the patient's eyes within the video. This step is crucial as it focuses on gathering detailed information from the eyes, which would be further used to detect and monitor existing medical conditions. The software then records data points from the video of the pupil's movement. The data points are used to analyze a patient’s medical conditions. The software calculates and generates a graph based on the left and right eye beats, further measuring the SPV value.

This visual representation helps clinicians in identifying patterns or anomalies. A report is sent to a clinician, who reviews the information. The clinician will add notes or observations based on the graph and recorded data, and the information will be saved in EHRs. A separate, more detailed report is created by the clinician, summarizing the patient's progress and recommending the next steps for management. Considering the framework findings and the clinician’s expertise, this would be a comprehensive assessment. Finally, the clinician sends the progress report and the management plan directly to the patient's mobile application. This ensures that the patient is kept in the loop and can access their health data and follow-up actions on their device.

The framework was utilized to capture a video of the subject using a smartphone and upload it to a cloud-based software application. The software application had an AI-based algorithm model that calculated data points of the eyes. Those data points were then converted to beats per second, and a graph was generated. The graph was then analyzed by an audiologist through a telehealth application and compared with the patient's history through EHRs. The interpretations were made, and the audiologist entered patient notes, which were then formed as reports and saved to the EHR. A separate report was generated, which included the next steps of management, and the report was sent to the subject. After a period, the patient had to re-record and upload the video on the application. The audiologist would be able to monitor the progress. It would allow the comparison of the diagnosis with past results, generate a progress report for the patient, and suggest the following management steps.

**Figure 2:**
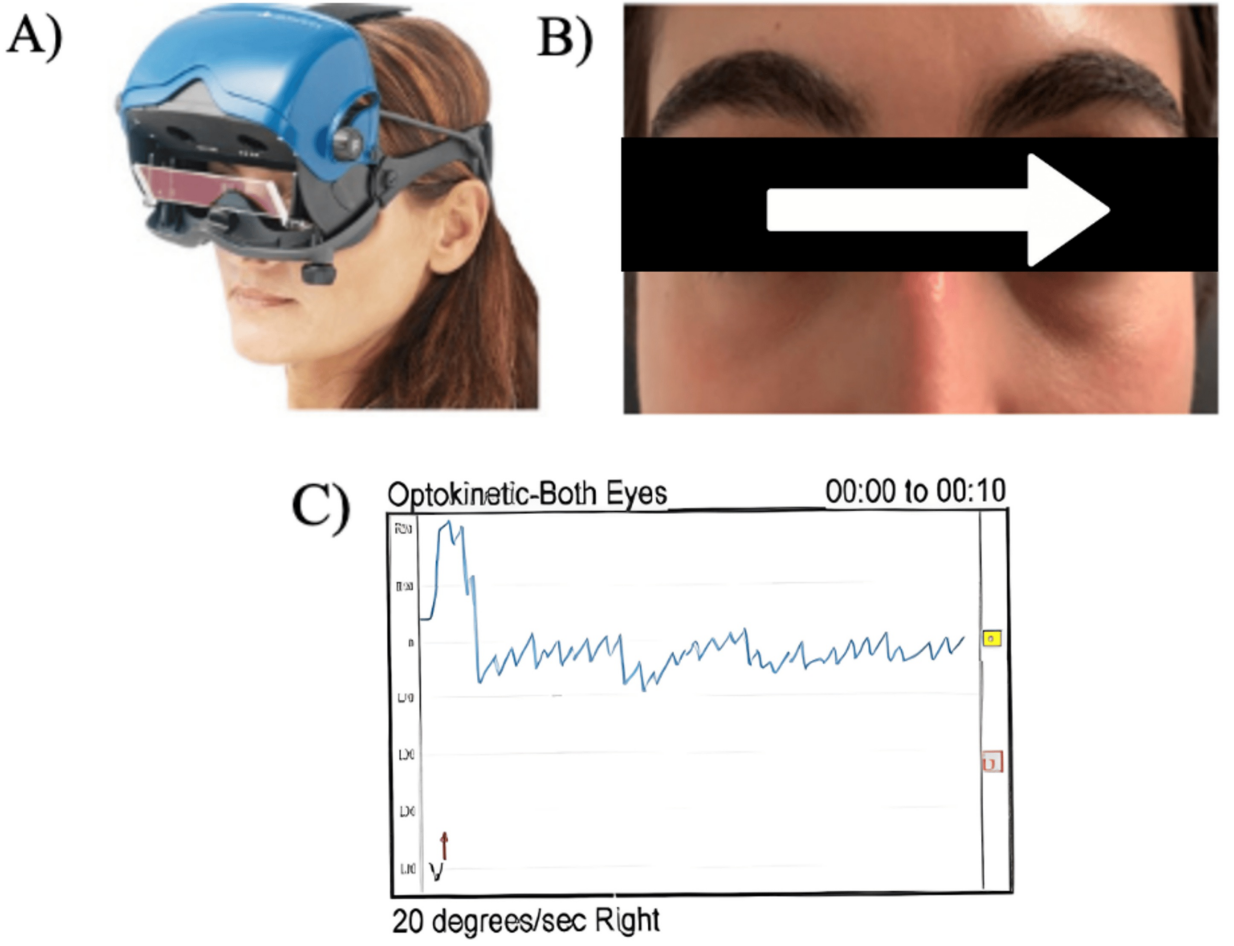
VNG device positioned on the patient’s head, displaying the visual stimulus and corresponding eye movement graph (A) Patient wearing the videonystagmography (VNG) headset, demonstrating the standard clinical setup used to detect and measure nystagmus. (B) Close-up view showing the direction of eye movement tracked by the VNG machine. (C) Graphical representation of recorded eye movements, specifically the slow-phase velocity (SPV) in degrees per second, generated by the VNG software.

**Figure 3:**
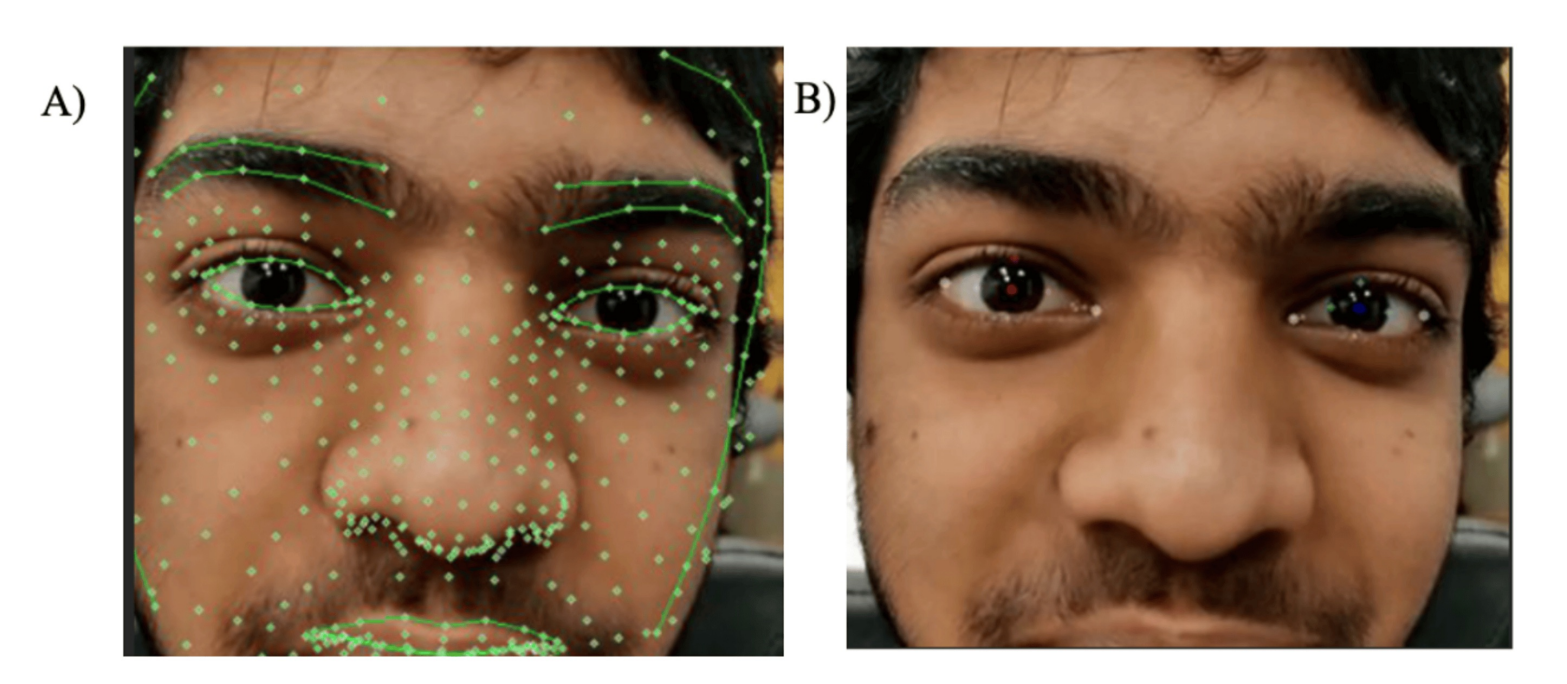
Mesh grid on the subject’s face through AI-based algorithm (A) Volunteer model's face with a detailed mesh overlay generated by the AI-driven algorithm, highlighting 468 distinct facial landmarks identified in real time. (B) Original image of the volunteer's model face used for the AI analysis without the mesh, providing context for the precise tracking performed by the system. Note: Written informed consent to include these images in an open-access article was obtained from the volunteer.

The technology stack includes Python, which is employed to track eye movements. MediaPipe’s Face Mesh is a machine learning solution to detect facial landmarks in real time. OpenCV is a library to manipulate images and video feeds. It starts by initializing the Face Mesh object with the parameter max_num_faces = 1 (meaning only one face will be detected), refined_landmarks = True (improves the precision of the landmarks), and minimum confidence levels for detection and tracking. The frames are resized to 1500 × 900 pixels and their color scheme changed to RGB using OpenCV’s function. For each frame in the video, face landmarks are processed with face_mesh.process(), and the output is checked. If landmarks are detected, the plotting of eyes in the frame proceeds. If no landmarks are detected, it simply goes to the next frame. The detected landmarks are first converted from relative coordinates to actual frame coordinates. The centers of the right and left irises are then calculated, and the values are stored. Then, key points around the left and right eye iris and corners are identified and plotted. Figure 2 represents the gold standard process utilized in audiology clinics. Figure 3 illustrates the mesh grid on the subject's face, which recognizes 468 3D facial marks through an AI-based algorithm. 

After processing all the landmarks, the script uses these landmarks to create a mask on the original frame. This mask is a transparent overlay where landmarks were found. Then, processed frames are plotted. "q" is used to break the loop or to stop/end the video. MediaPipe's Face Mesh is a machine-learning solution for high-fidelity facial geometry, including 468 3D facial landmarks that can track the full face down to the lips, including the eyebrows, eyes, nose, and other features. This model is built on TensorFlow. It is a lightweight model trained to detect facial landmarks using a large annotated dataset. It starts by using a machine learning model to detect the presence and location of a face in the image. Once the face has been detected, another model is used to identify the 468 distinct landmarks across the face. This model uses a mesh triangulation approach to pinpoint different facial features. One of the key features of this model is that it does not need depth cameras or other special hardware. It operates purely on 2D images and uses machine learning to infer the 3D structure of the face.

Gaze angles for the person’s right and left eyes are determined using the iris coordinates detected by MediaPipe. The pupil width is first calculated by subtracting the right and left corners of the left and right eyes. The distance between the eye's center and the pupil's center is then computed for each frame. Since the eye radius is a constant value of 11.5 mm across humans, this measurement is used to estimate the gaze angle. Assuming that the eye is a sphere, a formula has been designed to calculate the angle.

To develop the model, the dataset is split into a training set and a test set, with 80% of the data used for training and the remaining 20% reserved for testing. A linear regression model is then created and trained using polynomial features. Model evaluation is performed by comparing the predictions on the test set with the actual values, computing the R-squared score, which measures how well the model fits the data. A high R-squared value indicates a better fit. Once trained, the model makes new predictions on raw data. Finally, the trained model is saved using the pickle library, allowing it to be loaded and used later without retraining.

Equation 1 is significant for tracking eye movement. It translates the pupil's position into an angular measure, which can be used to analyze gaze direction or nystagmus patterns. Using the eye circumference and degrees, this approach provides a consistent and scalable way to quantify eye movements across different individuals and scenarios. Such calculations can help detect and diagnose conditions like nystagmus, strabismus, or other eye movement disorders in clinical settings. The formula could be used in AI-based eye tracking systems to process video data and compute eye movement angles for analysis and reporting.

The proposed method introduces an innovative telemedicine framework for analyzing patient eye movements with high accuracy and efficiency. 

Study design

This study was conducted at Labyrinth Audiology, Boca Raton, US. The methodology of this study involves a structured train-test-validation split and detailed performance metrics to ensure the robustness of the proposed model. The dataset consists of 15,000 frames divided into a 70:20:10 ratio for training, testing, and validation. This distribution allows for a comprehensive evaluation of model performance across unseen data, minimizing overfitting and improving generalization.

The performance metrics indicate that the model reliably predicts outcomes, demonstrating its overall effectiveness. The model achieves a high accuracy of 98%, showcasing its precision in detecting and analyzing nystagmus. The mean squared error (0.00459) is notably low, indicating precise predictions and highlighting the model's stability and reliability. Furthermore, the correction error margin (±4.8%) confirms the model’s robustness under real-world conditions, ensuring consistent performance. The statistical significance of the results, with p < 0.05, validates that the findings are unlikely to be due to chance, reinforcing the reliability of the proposed AI-driven framework.

Model Analysis Through Calibration Polynomials

As Rosengren et al. described, gaze estimation is also known as point of regard (PoR), which is calculated for computer-based diagnostics [44]. The PoR estimation is calculated by $pPoR = [xPoR yPoR]^{T}$, which is computed using a polynomial (P) and eye tracker data (uPC), where $uPC = [xPC yPC]^{T}$. The selective structure of P determines the structure of uPC. The main aim of the calibration is to estimate the coefficients of the polynomial.

Equation 2, $pPoR=PuPC$, is used to establish the initial or default state of the pupil. When measuring angular displacements, the pupil center can serve as the origin from which movements are calculated. In systems analyzing eye movements (e.g., for diagnosing nystagmus or gaze tracking), this equation might define the origin or default state of the pupil for comparisons, where ph and pv are horizontal and vertical polynomials, respectively. The coefficients are estimated using a least squares solution.

In Equation 3, $P=[\binom{P_{h}}{P_{v}}​​]$, the vector form is concise and allows easy manipulation in mathematical operations, such as transformations or projections. This equation is likely used to represent the pupil's position in 2D space during eye movement analysis, where d is either the horizontal or the vertical direction and UPC is the matrix containing the calibration data vectors for each calibration target.

Equation 4, $pd=(U_{PC}U^{T}_{PC}​)^{-1}U^{T}_{PC}t_{d}$​, is typical in projection operations, such as in regression, machine learning, or signal processing, where data is transformed into a specific feature space. Td is a vector with calibration targets of direction d, and n is the number of calibration targets.

Equation 5, $UPC​=[uPC​(1)&hellip;uPC​(n)]$, explains​ the capturing of a series of measurements, enabling the analysis of changes in the pupil center over time or across samples. This matrix/vector can serve as input for transformations, projections, or statistical models that require data in a structured form. The representation is suitable for linear algebra operations (e.g., matrix multiplication, and inversion), often used in signal processing, eye tracking systems, or motion analysis.

Equation 6, $A_{1}=\left[\binom{a_{0,c}}{a_{1,c}} \binom{a_{0,x}}{0} \binom{0}{a_{1,y}} \right]$, might transform input data (e.g., from pupil measurements) into a different coordinate system or space for further analysis. Such a matrix is commonly used to model eye movements, enabling the decomposition or reconstruction of positions and directions.

Equation 7, $B=\left[\binom{b_{0,c}}{b_{1,c}} \binom{b_{0,x}}{b_{1,y}} \binom{b_{0,y}}{b_{1,y}}\binom{b_{0,xy}}{b_{1,xy}} \right]$, is suitable for systems that analyze multidimensional data (e.g., eye movement) and combine various components into a compact representation. This matrix could be part of a system that processes eye movement data, capturing individual (x, y) and combined (xy) effects to better model the gaze direction or pupil motion.

Equation 8 represents a matrix in eye tracking, which can model linear and nonlinear effects of gaze direction, pupil movement, or eye dynamics. In the equation below, the structure accommodates data with higher complexity, making it suitable for systems that analyze intricate motion patterns or interactions between dimensions.

\(g = \begin{bmatrix} g_{0,c} & g_{0,x} & g_{0,y} & g_{0,x^2} & g_{0,y^2} & g_{0,xy} \\ g_{1,c} & g_{1,x} & g_{1,y} & g_{1,x^2} & g_{1,y^2} & g_{1,xy} \end{bmatrix}\)

Equation 9 represents the matrix that could be used in eye tracking systems to model advanced gaze dynamics, including nonlinear effects that occur in natural eye movements. It serves as a foundation for transformations that require capturing detailed patterns and dependencies in multidimensional data, as given below. \(A_4 = \begin{bmatrix} a_{0,c} & a_{1,c} \\ a_{0,x} & 0 \\ a_{0,x^2} & 0 \\ a_{0,x^3} & 0 \\ a_{0,x^4} & 0 \\ 0 & a_{1,y} \\ 0 & a_{1,y^2} \\ 0 & a_{1,y^3} \\ 0 & a_{1,y^4} \end{bmatrix}\)

## Results

The nystagmus beats on a time scale is a plot showing eye movements over time, which can help identify the presence of nystagmus. The graph shows rapid oscillations of eye positions measured in degrees against a time scale in seconds. In nystagmus, such a graph would typically visualize the characteristic eye movements associated with the condition. Nystagmus can be identified by the rapid, uncontrollable movements of the eyes, which could be horizontal, vertical, or rotary. The frequency and amplitude of these movements can be crucial for diagnosis and could be depicted in the graphs provided. Eyeblinks were detected as spikes and removed by the algorithm. They are considered eyeblinks and will be removed by the algorithm as they do not have any clinical value. By calculating SPV, which is automatically calculated by the algorithm, the physicians can decide the rate of nystagmus. They can guide the next steps in treatment. The graph would be used by healthcare professionals to assess the severity of nystagmus and to monitor changes over time, which can be critical for determining the effectiveness of treatment or understanding the progression of an underlying condition. The graph generated by the proposed novel solution was then validated against the VNG machine output by a clinician, and a p < 0.05 was calculated through a statistical test method.

The sample size distribution used in the study categorizes the participants into two groups: those with nystagmus (N) and those without nystagmus (NN). Each group consists of 10 individuals, ensuring equal representation in the sample. This balanced distribution allows for a comparative analysis between the two groups in the research context. The subjects are geographically from the United States.

**Figure 4:**
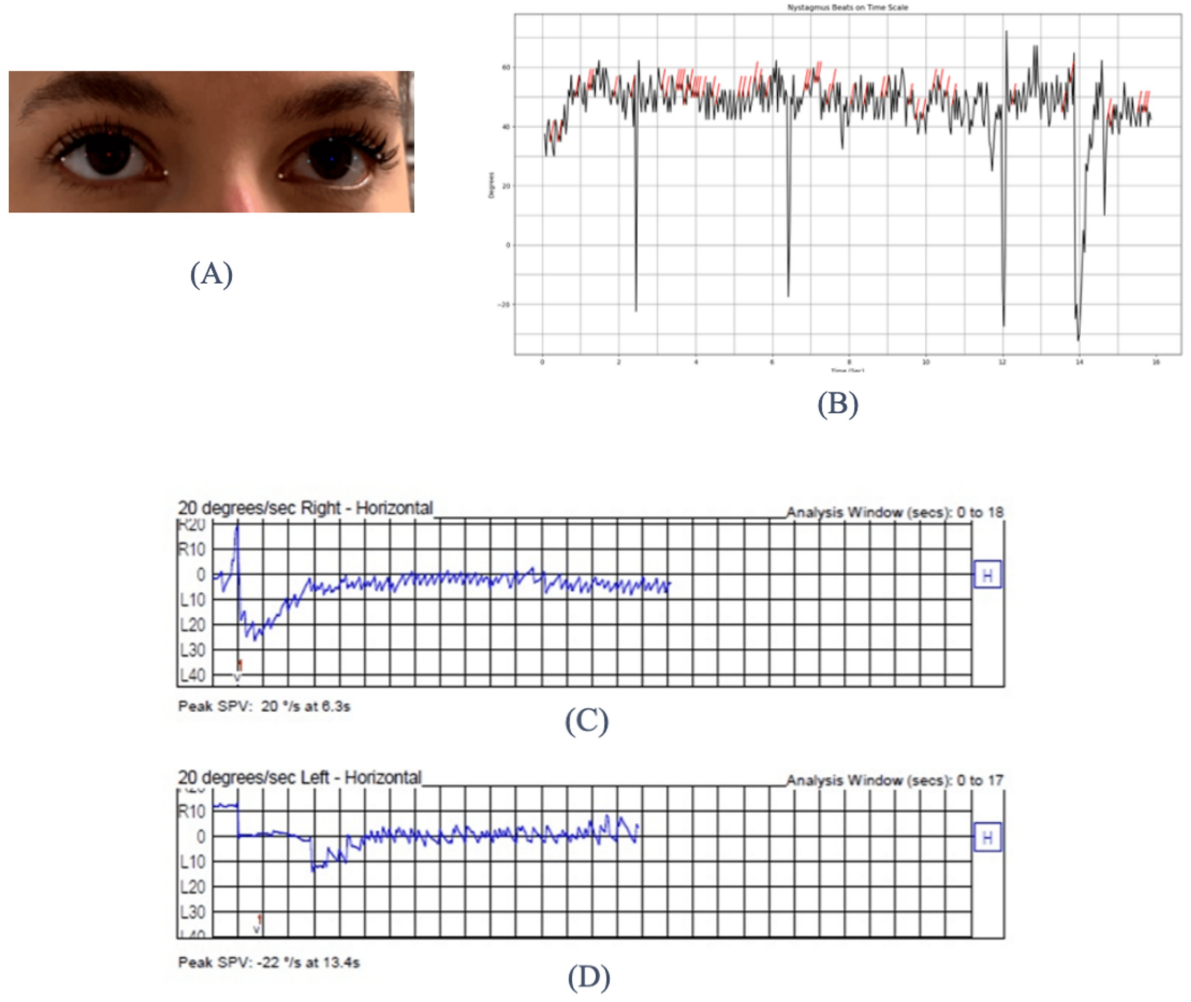
Comparison of the accuracy of results: data collected from Participant 1 using the AI-driven software and the VNG machine (A) Detection of eye movement data points by the AI-based algorithm. (B) Graph generated by the AI-driven software, visualizing eye movement velocity (in degrees per second (deg/s)) over time. The black line represents recorded eye movements, while the red overlay line specifically highlights rapid eye movements of nystagmus (fast-phase component). This visualization helps clinicians distinguish between slow-phase and fast-phase movements, which is critical for diagnosing the presence and type of nystagmus. (C) Graph produced by the standard videonystagmography (VNG) machine, showing comparable eye movement velocity measurements over the same period and serving as the gold standard. The slow-phase velocity (SPV), a key clinical indicator of nystagmus severity, is derived from this graph. Elevated SPV values generally correlate with more significant vestibular system dysfunction and help guide clinical decision-making regarding treatment urgency and type. Note: Written informed consent to include this image in an open-access article was obtained from the participant.

Figure 4 presents a comparison of Participant 1's optical penetrating keratoplasty (OPK) recording as captured by the VNG machine and the AI-driven system.

The Y-axis, labeled "Deg/s," represents the velocity of the eye movements in degrees per second. The numerical values range from -20 to 60, indicating the speed of the eye’s movement as it oscillates back and forth. The X-axis, labeled "Time (Sec)," represents time in seconds, ranging from 0 to approximately 16 seconds, providing the timeline over which the eye movements are recorded. The line plot, shown in black, oscillates above and below a baseline, characteristic of nystagmus waveforms that depict the velocity of eye movements over time. These oscillations represent the back-and-forth beating pattern typical of nystagmus. Additionally, a red overlay appears at various points on the black line plot, indicating a filtered signal or a specific eye movement component, such as quick phases or a distinct type of nystagmus beat that the analyst aims to highlight.

**Figure 5:**
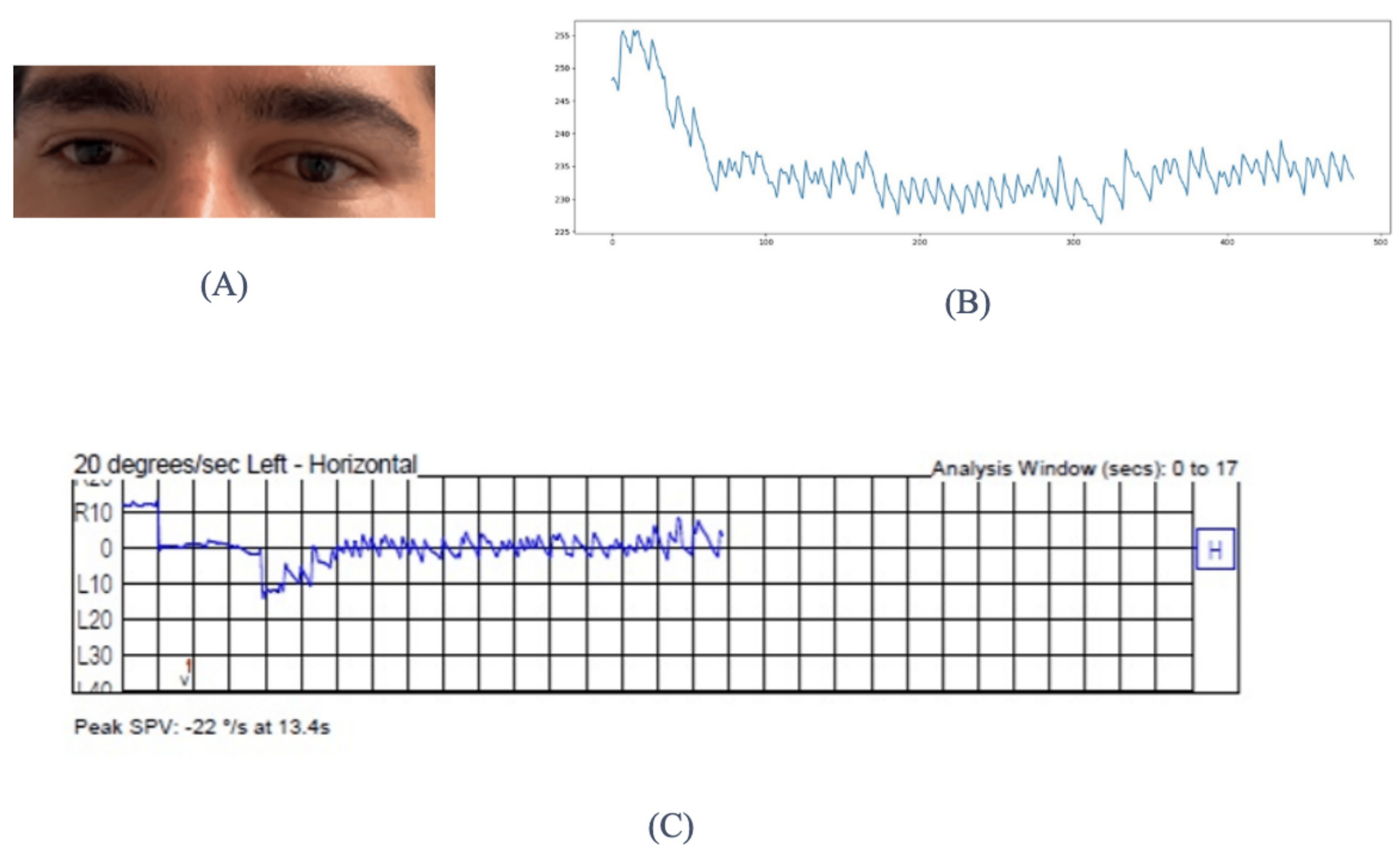
Comparison of the accuracy of results: data collected from Participant 2 using the AI-driven software and the VNG machine (A) Eye movements captured via smartphone camera for analysis by the AI-driven software. (B) Graph from the AI-driven software showing horizontal eye position relative to a neutral center point over an extended duration. The red line indicates horizontal eye position changes, reflecting repetitive leftward and rightward movements characteristic of horizontal nystagmus. The peak slow-phase velocity (SPV) value recorded by the AI software (20 deg/s) represents the fastest velocity during slow-phase eye movement, a key clinical measure for assessing nystagmus severity and directionality. (C) Graph from the videonystagmography (VNG) machine similarly depicting horizontal eye movements, serving as the standard clinical reference. Its recorded SPV value of 22 deg/s closely matches the AI-calculated SPV, demonstrating strong diagnostic agreement. These measurements are essential for clinicians in diagnosing the precise vestibular disorder (such as benign paroxysmal positional vertigo), assessing its severity, and guiding patient management decisions. Note: Written informed consent to include this image in an open-access article was obtained from the participant.

Figure 5 presents a comparison of Participant 2's OPK recording as captured by the VNG machine and the AI-driven system.

The X-axis represents the analysis window in seconds, ranging from 0 to 500 seconds, indicating the duration over which eye movements are tracked. The Y-axis is labeled with values such as R10, L10, R20, L20, R30, and L30, denoting eye position in degrees relative to the center (0), with "R" indicating correct eye positions and "L" indicating left eye positions. The red line in the graph represents eye movement data, with fluctuations indicating changes in eye position as it moves horizontally. The fact that the red line remains predominantly below the center suggests a consistent leftward movement. The peak SPV is also noted as 20 deg/s, a key measurement used to characterize nystagmus. This indicates that the fastest recorded movement, specifically during the slow phase of nystagmus, was 20 deg/s.

The initial drop could indicate a rapid change in eye position, while the subsequent fluctuations might represent the back-and-forth movement characteristic of nystagmus. Such a graph is utilized to assess eye tracking and stability. In this context, it would help to diagnose the presence and characteristics of nystagmus, informing the clinician about its type (e.g., jerk or pendular), velocity, and potentially its etiology. The SPV value calculated by the VNG machine was 22 deg/s, and the value calculated by the AI-driven software was 20 deg/s.

Data points of eye movement generated from the software were converted to beats per second, a graphical representation was generated, and the SPV value was determined. The SPV value was verified using the VNG machine graphs and by the clinician for statistical significance. The average value of the SPV was compared to the value generated by the VNG machine and compared. The calculated statistical value is p < 0.05, the mean squared error is 0.00459, and the correction error is ±4.8%, which shows the high accuracy of the framework, and the model was validated. Conditions such as BPPV can be treated with canalith repositioning. This algorithm checked and validated the pre- and post-treatment changes in nystagmus.

The proposed model was developed, and the value of SPV was calculated. The mathematical formulations were calculated, and then the physician could decide the rate of nystagmus and assist the patient with the following management steps.

## Discussion

Numerous application areas in the healthcare industry use digital twin models designed with AI, including neurology [42,43], where conditions such as autism [44] and stuttering [45] are addressed. The computational platforms on which these applications are deployed are configured using a Graphical User Interface [46] based on high computational performance. In medicine, there are a few other areas where the applications of AI are prominent, including orthopedics [47] and ophthalmology [48], which include robotic systems for disinfection [49]. In audiology, we developed the proposed framework, which assists the audiologist through biofeedback analysis in making consultation available to people at a remote location through a secured healthcare system [50]. Patients benefit from convenient telemedicine visits, reducing travel and expenses. Physicians gain flexibility with the clinical or virtual use of the model. To some extent, it can replace portions of traditional methods like VNG and ENG, reducing costs and improving efficiency.

This technological advancement transforms how healthcare professionals diagnose and treat patients [46,47]. Deep learning models can assist in diagnosing more patients and referring them to the correct medical specialist for treatment, as well as assisting physicians in following up with patients post-treatment. The patient’s needs, therefore, are addressed by giving them accessibility, convenience, and savings by meeting with their healthcare provider through a telemedicine visit [47]. The model also provides a cost benefit to healthcare providers by giving them the flexibility of using this model in a clinical or virtual setting compared to implementing VNG (inner ear testing) or ENG (inner ear vestibular assessment using infrared goggles) in an actual office setting [48]. This biofeedback analysis assisted the audiologist in making consultation available to people situated at a remote location.

Clinical use of the proposed framework

The proposed framework integrates telemedicine and AI, providing a novel approach to detecting and analyzing nystagmus remotely. By enabling a caregiver to record videos of a patient’s eye movements using a smartphone and upload them to a cloud-based platform, the system reduces the need for in-person visits, making eye movement diagnostics more accessible, particularly for patients in remote or underserved areas who may not have immediate access to specialized neuro-ophthalmologists or audiologists. This telemedicine-driven approach enhances accessibility while ensuring timely diagnosis and management of vestibular and neurological disorders associated with nystagmus.

The system features automated evaluation, where the AI-driven software analyzes eye movement videos, calculates beats per second, and generates a graph that provides a quantitative visual summary of the patient’s SPV and other critical parameters [50]. This automation eliminates subjective variability, ensuring standardized and reproducible results that physicians can use to guide their clinical decisions. The physician-focused workflow ensures that while the model automates data collection and preliminary analysis, the final interpretation remains clinician-driven. The physician reviews the AI-generated report, validates the SPV values, and determines the next steps for patient management, ensuring that AI is an assistive tool rather than a replacement for clinical expertise.

One of the key advantages of this framework is its high accuracy and reliability, achieving 98% accuracy with a mean squared error of 0.00459 and a correction error margin of ±4.8%, confirming its robustness for real-world applications. This precision is crucial in telemedicine, where diagnostic reliability must be comparable to in-clinic VNG to ensure confidence in remote evaluations [46]. The framework follows an end-to-end pipeline, streamlining the entire process from video capture to graph generation and, ultimately, physician evaluation. By providing a fully integrated telehealth-compatible model, the system ensures seamless workflow integration for primary care physicians, neurologists, and otolaryngologists, offering a cost-effective and scalable alternative to traditional nystagmus detection [49].

Finally, the framework is scalable for real-world use, as evidenced by its training on 15,000 frames with a well-balanced 70:20:10 train-test-validation split. This structured data-splitting strategy ensures the model’s generalizability across diverse patient populations, reducing the risk of overfitting while maintaining high diagnostic fidelity. The ability to remotely capture, process, and analyze eye movements through AI-driven automation makes this framework a transformative tool for telemedicine, offering an accessible, cost-efficient, and clinically validated solution for diagnosing and monitoring nystagmus and other vestibular disorders in urban and rural healthcare settings.

The proposed AI-driven telehealth system is particularly effective in detecting horizontal nystagmus, as indicated by the study's emphasis on tracking horizontal eye movements and quantifying SPV. The model’s capability was primarily demonstrated with horizontal eye movement data, suggesting superior diagnostic accuracy and applicability for horizontal forms of nystagmus compared to torsional or vertical types.

The system is designed to be versatile for implementation in various clinical environments, including emergency departments, primary care settings, and audiology clinics. The discussion explicitly mentions its usefulness in tele-neurology scenarios within emergency departments for rapid initial screening, primary care practices for routine evaluation, and audiology clinics for detailed diagnostic assessments and ongoing patient monitoring.

This study represents a preliminary proof-of-concept, and the authors clearly outline their intentions to pursue further clinical trials to validate the framework’s performance more broadly. Future efforts will focus on expanding the sample size and diversity and refining the model’s accuracy and reliability under varied clinical conditions. These steps are crucial as the authors progress toward obtaining FDA approval to ensure broader clinical acceptance and real-world applicability.

However, the small validation sample size, limited participant diversity, challenges associated with motion artifacts and lighting variations, and constraints related to patient confidentiality restricting dataset availability limit the study's applicability in the real world. Therefore, the future goal of this study is to take it for further clinical trials through the FDA approval process. The framework in this paper can be used in mission clinics to screen patients, and the cost is reduced significantly. The framework creates a remote screening opportunity for patients, which reduces their overall logistical expenses. The future study will have a much larger dataset, which a diverse patient population will also classify.

Limitations

The authors acknowledge that a primary limitation of the study is the small and non-diverse sample. With only 10 subjects per group (those with and without nystagmus), the findings may not generalize to broader populations or reflect the variability encountered in clinical practice. Additionally, all participants were geographically limited to the United States, limiting the study results' representativeness. Another noted limitation pertains to variability in the video quality used for analysis. The authors explicitly highlight that factors such as lighting conditions and patient movement can significantly impact the quality of video recordings. Such variability could affect the precision and reliability of the AI-driven analysis, introducing inconsistencies in the diagnostic outcomes. Lastly, the study highlights the inability to share the dataset publicly due to strict patient privacy regulations. This restriction limits the external validation and reproducibility of the results by independent researchers, which is essential for rigorous scientific verification and broader acceptance of the proposed telehealth framework.

## Conclusions

Preliminary results from integrating a deep learning model into a headset FOR nystagmus detection demonstrate promising potential. While the system successfully identifies nystagmus under controlled conditions, further training and refinement are needed to improve its accuracy and reliability. Factors such as patient variability, sensor noise from the headset, and limited dataset diversity may affect the model’s performance. As the model undergoes continued training on larger and more diverse datasets, along with algorithmic fine-tuning, the system could evolve into a valuable tool for non-invasive, real-time monitoring of nystagmus in clinical settings. However, further validation and testing are required before it can be considered a reliable diagnostic aid in routine medical practice.

## References

[1] Gangwani D, Sanghvi HA, Parmar V, Patel RH, Pandya AS (2023). A comprehensive review on cloud security using machine learning techniques. Artificial Intelligence in Cyber Security: Theories and Applications.

[2] Singh S, Banoub R, Sanghvi HA, Agarwal A, Chalam KV, Gupta S, Pandya AS (2024). An artificial intelligence driven approach for classification of ophthalmic images using convolutional neural network: an experimental study. Curr Med Imaging.

[3] Gill GS, Tsai J, Moxam J, Sanghvi HA, Gupta S (2024). Comparison of Gemini Advanced and ChatGPT 4.0's performances on the ophthalmology resident ophthalmic knowledge assessment program (OKAP) examination review question banks. Cureus.

[4] Sanghvi HA, Patel RH, Agarwal A, Gupta S, Sawhney V, Pandya AS (2023). A deep learning approach for classification of COVID and pneumonia using DenseNet-201. Int J Imaging Syst Technol.

[5] Chlebus G, Schenk A, Moltz JH, van Ginneken B, Hahn HK, Meine H (2018). Automatic liver tumor segmentation in CT with fully convolutional neural networks and object-based postprocessing. Sci Rep.

[6] Wang S, Yang DM, Rong R, Zhan X, Xiao G (2019). Pathology image analysis using segmentation deep learning algorithms. Am J Pathol.

[7] Jiang Y, Yang M, Wang S, Li X, Sun Y (2020). Emerging role of deep learning-based artificial intelligence in tumor pathology. Cancer Commun (Lond).

[8] Sanghvi HA, Gangwani D, Mohamed AA, Gajjar P, Patel OP, Pandya AS (2024). Revolutionizing patient care: the synergy of IoT and machine learning in smart healthcare. Advances in Computers.

[9] Wolfgang Wolfgang, Kelly Kelly (2019). Artificial intelligence and machine learning: pushing new boundaries in hearing technology. The Hearing Journal.

[10] Grassmann F, Mengelkamp J, Brandl C (2018). A deep learning algorithm for prediction of age-related eye disease study severity scale for age-related macular degeneration from color fundus photography. Ophthalmology.

[11] Blain M, Kassin MT, Varble N (2021). Determination of disease severity in COVID-19 patients using deep learning in chest X-ray images. Diagn Interv Radiol.

[12] Erdaş ÇB, Sümer E, Kibaroğlu S (2023). Neurodegenerative diseases detection and grading using gait dynamics. Multimed Tools Appl.

[13] Erdaş ÇB, Sümer E, Kibaroğlu S (2021). Neurodegenerative disease detection and severity prediction using deep learning approaches. Biomedical Signal Processing and Control.

[14] Strupp ML, Straumann D, Helmchen C (2021). Nystagmus: diagnosis, topographic anatomical localization and therapy. Klin Monbl Augenheilkd.

[15] Stahl JS, Averbuch-Heller L, Leigh RJ (2000). Acquired nystagmus. Arch Ophthalmol.

[16] Eggers SD, Bisdorff A, von Brevern M (2019). Classification of vestibular signs and examination techniques: nystagmus and nystagmus-like movements. J Vestib Res.

[17] Straube A, Bronstein A, Straumann D (2012). Nystagmus and oscillopsia. Eur J Neurol.

[18] Lee AG, Brazis PW (2006). Localizing forms of nystagmus: symptoms, diagnosis, and treatment. Curr Neurol Neurosci Rep.

[19] Bhattacharyya N, Baugh RF, Orvidas L (2008). Clinical practice guideline: benign paroxysmal positional vertigo. Otolaryngol Head Neck Surg.

[20] Choudhuri I, Sarvananthan N, Gottlob I (2007). Survey of management of acquired nystagmus in the United Kingdom. Eye (Lond).

[21] Moideen A, Konkimalla A, Tyagi AK (2023). Cross-sectional analysis of videonystagmography (VNG) findings in balance disorders. Cureus.

[22] Gupta SK, Mundra RK (2015). Electronystagmography a very useful diagnostic tool in cases of vertigo. Indian J Otolaryngol Head Neck Surg.

[23] Palmeri R, Kumar A (2025). Benign paroxysmal positional vertigo. StatPearls [Internet].

[24] Ganança MM, Caovilla HH, Ganança FF (2010). Electronystagmography versus videonystagmography. Braz J Otorhinolaryngol.

[25] Eremin IE, Fedtsov AV, Shova NI, Mikhailov VA (2023). A videonystagmography device using a commercial webcam. Biomed Eng.

[26] Wagle N, Morkos J, Liu J (2022). aEYE: a deep learning system for video nystagmus detection. Front Neurol.

[27] Punuganti SA, Otero-Millan J (2020). Detection of saccades and quick-phases in eye movement recordings with nystagmus. ACM Symposium on Eye Tracking Research and Applications.

[28] Wei K, Yang Q, Yang X, Liu Z (2022). Application of a pupil tracking method based on Yolov5-Deeplabv3+ fusion network on a new BPPV nystagmus recorder. International Conference on Biomedical and Intelligent Systems.

[29] Bastani PB, Rieiro H, Badihian S (2024). Quantifying induced nystagmus using a smartphone eye tracking application (EyePhone). J Am Heart Assoc.

[30] Friedrich MU, Schneider E., Buerklein M (2023). Smartphone video nystagmography using convolutional neural networks. J Neurol.

[31] Hirko KA, Kerver JM, Ford S, Szafranski C, Beckett J, Kitchen C, Wendling AL (2020). Telehealth in response to the COVID-19 pandemic: implications for rural health disparities. J Am Med Inform Assoc.

[32] Esteva A, Chou K, Yeung S (2021). Deep learning-enabled medical computer vision. NPJ Digit Med.

[33] von Martial R, Leinweber C, Hubert N, Rambold H, Haberl RL, Hubert GJ, Müller-Barna P (2021). Feasibility of telemedical HINTS (head impulse-nystagmus-test of skew) evaluation in patients with acute dizziness or vertigo in the emergency department of primary care hospitals. Front Neurol.

[34] Li H, Yang Z (2023). Torsional nystagmus recognition based on deep learning for vertigo diagnosis. Front Neurosci.

[35] Li H, Yang Z (2023). Vertical nystagmus recognition based on deep learning. Sensors (Basel).

[36] Kong S, Huang Z, Deng W, Zhan Y, Lv J, Cui Y (2023). Nystagmus patterns classification framework based on deep learning and optical flow. Comput Biol Med.

[37] Lee Y, Lee S, Han J, Seo YJ, Yang S (2023). A nystagmus extraction system using artificial intelligence for video-nystagmography. Sci Rep.

[38] Lu W, Li Z, Li Y (2022). A deep-learning model for 3d nystagmus detection and its primary application. The Lancet.

[39] Phillips JS, Newman JL, Cox SJ (2019). An investigation into the diagnostic accuracy, reliability, acceptability and safety of a novel device for continuous ambulatory vestibular assessment (CAVA). Sci Rep.

[40] Winnick AA, Chen CC, Chang TP, Kuo YH, Wang CF, Huang CH, Yang CC (2022). Automated nystagmus detection: accuracy of slow-phase and quick-phase algorithms to determine the presence of nystagmus. J Neurol Sci.

[41] Reinhardt S, Schmidt J, Schneider J, Schulte E, Schule E, Leuschel M, Schipper J (2023). Smartphone-based videonystagmography using artificial intelligence. Curr Dir Biomed Eng.

[42] Yiu YH, Aboulatta M, Raiser T, Ophey L, Flanagin VL, zu Eulenburg P, Ahmadi SA (2019). DeepVOG: open-source pupil segmentation and gaze estimation in neuroscience using deep learning. J Neurosci Methods.

[43] Pham TX, Choi JW, Mina RJ, Nguyen TX, Madjid SR, Yoo CD (2022). LAD: a hybrid deep learning system for benign paroxysmal positional vertigo disorders diagnostic. IEEE Access.

[44] Rosengren W, Nyström M, Hammar B, Stridh M (2020). A robust method for calibration of eye tracking data recorded during nystagmus. Behav Res Methods.

[45] Banoub RG, Sanghvi H, Gill GS (2024). Enhancing ophthalmic care: the transformative potential of digital twins in healthcare. Cureus.

[46] Gajjar P, Garg M, Desai S (2024). An empirical analysis of diffusion, autoencoders, and adversarial deep learning models for predicting dementia using high-fidelity MRI. IEEE Access.

[47] Patel K, Sanghvi H, Gill GS, Agarwal O, Pandya AS, Agarwal A, Gupta M (2024). Differentiating cystic lesions in the sellar region of the brain using artificial intelligence and machine learning for early diagnosis: a prospective review of the novel diagnostic modalities. Cureus.

[48] Alhalabi B, Sanghvi HA, Patel RH, Pandya AS, Torres EC (2022). A cloud based novel framework for addressing repetitive behavior in autistic individuals. 2022 IEEE World Conference on Applied Intelligence and Computing (AIC).

[49] Alhalabi B, Taylor J, Sanghvi HA, Pandya AS (2022). A proposed framework for stutter detection: implementation on embedded systems. 2022 IEEE World Conference on Applied Intelligence and Computing (AIC).

[50] Pandya SB, Sanghvi HA, Patel RH, Pandya AS (2022). GPU and FPGA based deployment of blockchain for cryptocurrency-a systematic review. International Conference on Computational Intelligence and Sustainable Engineering Solutions (CISES).

